# East London Hospital for Children, Shadwell

**Published:** 1903-05-16

**Authors:** 


### EAST LONDON HOSPITAL FOR CHILDREN, SHADWELL.

The festival dinner of the East London Hospital for Children and Dispensary for Women, Shadwell, took place on Monday, the 4th instant, the Hon. W. F. D. Smith, M.P., presiding. Some 60 guests sat down, of whom about one-third were ladies.

After the customary loyal toasts had been fittingly honoured, the chairman proposed "The East London Hospital for Children." He said a special interest attached to their festival dinner on that occasion because one of the founders of the institution died not many days ago, far away in Pretoria. This was Mrs. Heckford, who, with her husband, Dr. Heckford, in 1868, founded the East London Hospital. They had both volunteered for service as doctor and nurse in the cholera epidemic of 1866. When the epidemic was over they married, and although wealthy they devoted themselves to life in East London. They furnished an empty warehouse at Ratcliff Cross with ten beds, and that was the beginning of what is now one of the most beneficent hospitals in London. It was long since Mrs. Heckford took any part in the management, but the surroundings were much the same now as then. The district was so poor and the children were so ill-fed and weak that they had to be nourished before they could take advantage of the treatment the hospital afforded. A few figures would show how necessary it was in that district; but, unfortunately, although it was so necessary, it was to many people living in other parts of London almost as unknown as the central parts of Africa. It was probable that from week-end to week-end no man richer than an artisan ever passed their doors. Theirs was, indeed a great and good work. It had saved thousands of children's lives and was at present dealing with 1,700 in the wards every year, and if it did not save all their lives it did send back many of them to carry on their work restored to health and saved them from a life of sickness and suffering. But beside those 1,700 in-patients there were from 33,000 to 35,000 out-patients dealt with every year. And for all this the accommodation, both at Shadwell and at Bognor, was not sufficient, because in the last 20 years their out-patients alone had more than trebled in number, and to anybody in the least acquainted with hospital management it would be apparent that there must be a large number of cases which would find treatment within the wards if it were possible to find room for them. As a matter of fact they had only been able to increase the number of beds by five during that 20 years, and so it came about that they were forced to turn away cases which ought to be taken in. They knew that the last two years had been difficult years for those concerned with charitable institutions, and though new buildings were greatly needed it was impossible to find money for them while the annual income showed signs of decreasing rather than increasing. His visit had shown him not only what a blessing the hospital must be, but had also enabled him to realise how necessary another medical ward was and a ward for whooping cough; considerable improvements in the out-patient department were also needed, and, what perhaps struck him most of all, new kitchens and offices. They had the space there, the land was all ready, but the funds were wanting. And they asked not only for money, but for their personal interest. Those who could not subscribe much themselves could spread abroad the knowledge of how that institution was founded by two noble souls who sacrificed almost everything for love of the children. They could tell how the children at Bognor were nursed and nourished back to life again. He was not ashamed to beg for such a cause, and he asked them to make sacrifices in the confidence that they were all desirous to serve the sick and suffering children of East London.

The Earl of Errol replied. It was no small compliment, he said, to be entrusted with the response for an institution of which they were all so justly proud. But he could not help feeling that the statement of their Resident Medical Officer that "on a low computation some 300 medical cases, eminently suitable for admission, were rejected during the year for lack of room" constituted a shameful slur on our boasted civilisation, and was a standing reproach to one of the wealthiest cities of the world. Their assured income was under £1,000 a year, while their expenses were nearly £10,000. It always seemed to him something to be ashamed of that in a great wealthy community hospitals should have to beg for funds, and he could not help thinking—though it was a solution that he for one should be sorry to see—that if the public did not come forward more generously, the hospitals would have to be put on the rates. He believed there was a certain number of well-to-do people who gave little or nothing to hospitals. He should like to take such people down into Shadwell and show them round; and if they were not moved to do something then, he was very sorry for them and did not envy them their wealth.

Mr. Henry Samuel, M.P., proposed the health of the Board of Management and the Medical Staff, which was responded to by Colonel Needham, the Chairman, and Mr. Cuthbert Wallace, who observed that the relations of the staff with the lay board were as cordial as could be, their laws being such as might well be taken as an example by many institutions.

Other toasts followed, and the Secretary, Mr. Thomas Hayes, announced that the subscriptions amounted to £1,705, including one of £250 from the Chairman and £25 from his wife, Lady Esther Smith.

